# Sleep Is Associated with Offline Improvement of Motor Sequence Skill in Children

**DOI:** 10.1371/journal.pone.0111635

**Published:** 2014-11-05

**Authors:** Sho K. Sugawara, Satoshi Tanaka, Daisuke Tanaka, Ayumi Seki, Hitoshi T. Uchiyama, Shuntaro Okazaki, Tastuya Koeda, Norihiro Sadato

**Affiliations:** 1 Division of Cerebral Integration, National Institute for Physiological Sciences, Okazaki, Japan; 2 Research Fellow of the Japan Society for the Promotion of Science, Tokyo, Japan; 3 Laboratory of Psychology, Hamamatsu University School of Medicine, Hamamatsu, Japan; 4 Department of Education, Faculty of Regional Sciences, Tottori University, Tottori, Japan; 5 Department of Clinical Research, Tottori Medical Center, National Hospital Organization, Tottori, Japan; 6 School of Health Sciences, International University of Health and Welfare, Ohtawara, Japan; 7 Department of Physiological Sciences, The Graduate University for Advanced Studies (SOKENDAI), Hayama, Japan; Tokai University, Japan

## Abstract

In adults, sleep is necessary for the offline improvement of certain skills, such as sequential finger tapping, but whether children show a similar effect is still debatable. Here, we tested whether sleep is associated with offline performance improvement in children. Nine- and 11-year-old children trained on an explicit sequential finger tapping task. On the night following training, their parents observed and recorded the duration of each child’s sleep. The following day, all children performed a surprise retest session on the previously trained sequence. In both 9- and 11-year-old children, skill performance was significantly improved during the first retest session relative to the end of training on the previous day, confirming the offline improvement in performance. There was a significant correlation between the degree of improvement and sleep duration the night after training, suggesting that in children, as in adults, sleep is associated with offline skill enhancement.

## Introduction

Newly acquired skills become more robust and stable during the retention interval following the end of practice [1]–[4]. These offline changes take two different forms: enhancement and stabilization [4], [5]. Enhancement refers to the offline performance improvement that occurs without any physical practice. During stabilization, previously acquired skills become resistant to interference, such as new skill learning, over time. The offline improvement of implicit motor learning, such as implicit sequential learning and force-field adaptation, depends on the passage of time rather than sleep [3], [6], [7]. On the other hand, it is well-known that in healthy adults [7], [8] the consolidation of explicit sequential learning, such as sequential finger-tapping, strongly depends on sleep, specifically non-rapid eye movement (NREM) 2 sleep and sleep spindles [8]–[10].

In previous behavioral studies, 9- to 12-year-old children exhibited robust performance improvements in explicit motor sequence skill 24 hours after training [11],[12], although these studies did not measure any properties of the children’s sleep during the retention interval after training. However, previous studies have reported that this enhancement is not sleep-dependent in children [13]–[15]. The most recent study showed that sleep induced a significant offline improvement only when 4- to 6-year-old children performed extra practice prior to completing the standard amount of training [16]. Therefore, the effect of sleep on motor skill enhancement in children remains unclear. The purpose of this study was to investigate the correlation between sleep and skill consolidation, factoring in the children’s age and the type of motor-training task. Our hypothesis was that, if the mechanism by which sleep consolidates explicit motor sequence learning in children is the same as in adults, sleep duration should correlate with performance, because the proportionality of the architecture of sleep suggests that the longer the duration of total sleep, the greater the amount of NREM2 sleep.

To investigate this hypothesis, in the present study we evaluated the correlation between sleep duration the night after training and the offline performance improvement at retest. To control for the effect of the passage of time, the retention interval between training and retest was the same (24 hours) for all participants. This approach allowed us to test whether post-training sleep facilitates the enhancement of a trained skill in children who sleep in their natural sleep environment.

## Materials and Methods

### Participants

As the participants were minors, written informed consent was obtained from each participant and their parent prior to the experiment. The study was conducted according to the Declaration of Helsinki and approved by the Internal Review Board of Tottori University. Twenty-five children (14 males and 11 females, mean [M]±standard deviation [SD] = 9.48±1.16 years) participated in this study. Based on Edinburgh’s Laterality Quotient (LQ), one female was excluded from the analyses for left-handedness (LQ = −1.00). Thus, data from 24 right-handed children (14 males and 10 females; M±SD = 9.42±1.14 years; 14 9-year-olds and 10 11-year-olds; Edinburgh’s LQ, M ± SD = 0.89±0.23) were used for the analysis. Participants came to the laboratory on two subsequent days. None of participants had a history of neurological, psychiatric, or sleep disorders.

### Experimental procedure

All participants were trained on a modified version of a sequential finger-tapping task on day 1. The original version of the sequential finger-tapping task required participants to press four numeric keys on a standard computer keyboard repeatedly with the fingers of their non-dominant (left) hand as quickly and as accurately as possible for 30-s periods (for details, see [8], [17]). Given that finger-tapping speed depends on age [18], the modified version of this task required children to press three keys with their index, middle, and ring fingers. A white asterisk appeared on a computer monitor at one of three possible positions within an equally spaced horizontal array. Each of the three positions corresponded to one of three buttons on a numeric keyboard. Sequenced stimuli were presented repeatedly for 30 s. On day 1, participants trained on sequence A (“3-1-2-1-3”). After training, all participants received visual feedback about their performance (for example, their learning curve). On the following day, all participants performed a surprise test of the trained sequence. Because the expectation of a future test might motivate participants to mentally or physically rehearse the relevant skill, we used a surprise test to minimize the possibility of any mental or physical practice of the sequence after training.

Finger tapping performance was evaluated by the number of correctly tapped sequences per 30-s trial. The improvement in performance following a night of sleep was defined as the percent increase in mean performance between the last three trials during training on day 1 and the first three retest trials on day 2 [9], [17], [19]. Training on day 1 consisted of twelve 30-s trials with 30-s rest periods between trials, whereas the retest on day 2 consisted of only five trials with the same rest interval.

### Sleep duration and additional ratings

To examine the effect of sleep duration on the improvement in motor skill, participants’ parents were asked to observe and report the time that their child went to bed on the nights before and after training, and the time that they woke up on the training and retest mornings [19]. To measure sleep duration, we asked the participants’ parents two questions: “What time did your child go to sleep last night?” and “What time did your child wake up this morning?”. Sleep durations were calculated from these parental reports. To ensure that these times did not differ from the child’s usual sleep/wake times, parents also answered an additional question: “Was this sleep-wake cycle consistent for the past four weeks?”. All participants’ sleep/wake times were consistent with their usual sleep cycles, with the exception of two participants.

It was possible that participants’ subjective states during training or retest might influence their performance. Thus, at the end of the training and retest periods, all participants completed questionnaires providing their subjective ratings of alertness (1 = not at all sleepy, 10 = very sleepy, modified from [20]), concentration (1 = not at all focused, 10 = very focused [19]), and fatigue (1 = high level of fatigue, 10 = no fatigue, modified from [21]) during training and retest using a ten-point scale.

### Data analysis

Statistical analyses were based on the general linear model using analyses of variance (ANOVAs) for independent and repeated measures. Then, the degree of improvement was compared between the two age groups using unpaired t-tests (two-tailed). We also conducted multiple regression analysis to investigate the relationship between sleep and skill enhancement in children. Sleep duration during the night after training (in hours) was included as an independent variable to test our hypothesis that the duration of sleep is related to the degree of performance improvement. We also included participants’ age in the model, because performance on the motor task is known to be age-dependent [12]. We included the time intervals between wake-up and the performance of the retest (in hours) as an independent variable in order to evaluate the effect of fatigue resulting from the child being awake for a longer period of time prior to retest. To test for multicolinearity between these variables, we performed correlation analyses prior to the regression analysis, and confirmed that there were no significant correlations between these variables (**Table 1**). We report R-squared values as the coefficients of determination for the multiple regression analysis. All analyses were performed in SPSS 19.0 (SPSS Inc., Chicago, USA). For all analyses, the significance level was *p*<0.05. The data used for these analyses are available in **Table S1** and **Table S2 in File S1**.

**Table 1:** Correlation coefficients between demographic variables, subjective ratings, and sleep duration.

Variables	1	2	3	4	5	6	7	8	9	10	11	12	13	14	15	16
1. Age	–	−0.01	−0.16	0.28	0.34	−0.06	0.13	0.01	−0.04	−0.16	0.11	0.28	0.52**	0.13	−0.23	−0.04
2. Experiment onset time		–	−0.37	−0.13	−0.08	−0.25	0.01	−0.02	0.96**	−0.62**	−0.14	−0.42*	0.23	0.08	−0.09	0.97**
3. Sleepiness on day 1 (1–10)			–	0.21	0.30	−0.20	0.14	0.26	−0.40*	0.70**	−0.01	0.28	−0.01	0.25	0.16	−0.43*
4. Concentration on day 1 (1–10)				–	0.37	−0.08	0.13	−0.08	−0.17	0.41*	0.65**	0.45*	0.20	0.28	0.08	−0.2
5. Fatigue on day 1 (1–10)					–	−0.20	0.33	0.41*	−0.17	0.01	0.12	0.58*	−0.03	0.46*	0.33	−0.19
6. Sleep time 1						–	0.15	−0.18	−0.28	−0.05	−0.08	0.09	−0.24	0.07	0.20	−0.26
7. Wake-up time 1							–	0.76**	−0.18	0.01	−0.02	0.18	−0.04	0.65**	0.46*	−0.06
8. Sleep duration 1 (hours)								–	−0.23	0.06	−0.24	0.21	−0.17	0.55**	0.47*	−0.15
9. Awake duration 1 (hours)									–	−0.62	−0.13	−0.46*	0.24	−0.11	−0.22	0.98**
10. Sleepiness on day 2 (1–10)										–	0.43*	0.5*	−0.22	0.16	0.22	−0.66**
11. Concentration on day 2 (1–10)											–	0.46*	−0.16	0.17	0.23	−0.18
12. Fatigue on day 2 (1–10)												–	−0.23	0.24	0.33	−0.47*
13. Sleep time 2													–	−0.19	−0.74**	0.27
14. Wake-up time 2														–	0.79**	−0.17
15. Sleep duration 2 (hours)															–	−0.28
16. Awake duration 2 (hours)																–

*Note.* Sleep time 1 and 2 indicate the time that children went to bed on the nights before and after training, respectively. Wake-up time 1 and 2 represent the time that they woke up on the training and retest mornings, respectively. Sleep durations were calculated from the sleep times and wake-up times. Awake duration 1 and 2 represent the time intervals from wake-up to when children performed the training and the retest tasks. *p<0.05,

**p<0.01.

## Results

### Performance changes during new learning on day 1

**Figure 1:**
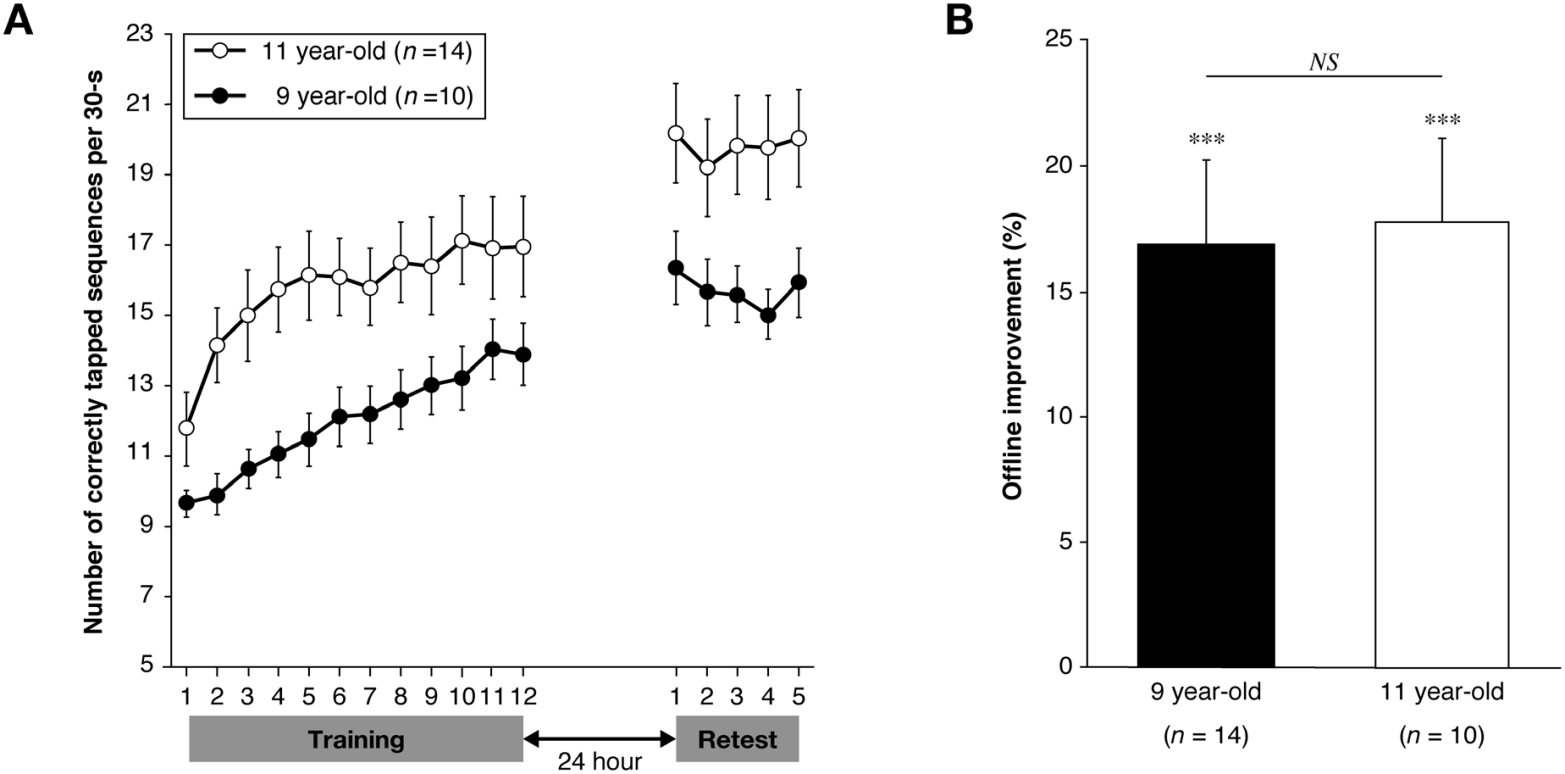
Motor sequence performance and improvement between days in the two different age groups. (**A**) Participants in both groups showed significant offline improvement, measured by the performance improvement between the last three trials during training on day 1 and the first three trials during retest on day 2 (*p* values<0.001). The black circles represent the mean performance during each trial in the 9-year-old group, and the open circles represent the mean performance in the 11-year-old group. (**B**) In total, performance in the 11-year-old children was significantly better than that in the 9-year-old children (*p*<0.05). However, the degree of offline improvement did not differ between the age groups (two-tailed unpaired t-test; *p* = 0.51). The black bar represents the 9-year-old group and the white bar represents the 11-year-old group. Error bars indicate the standard error of the mean (SEM). ***p<0.001; **p<0.01 (repeated-measures ANOVA).

To test whether there were differences between baseline performance and performance at the end of training between the groups, we conducted a 2 (group; 9- vs. 11-year old)×2 (learning; baseline vs. end of training) ANOVA. There was no significant interaction (ANOVA, *F*_2,22_ = 1.10, *p* = 0.31; **Fig. 1A**), but the main effects of group (*F*_1,22_ = 5.25, *p*<0.05) and learning (*F*_1,22_ = 71.13, *p*<0.001) were significant.

### Performance changes after new learning and between test days 1 and 2

Performance of the sequential finger-tapping task significantly differed between the groups, indicating that 11-year-old children showed better overall performance than the 9-year-old children (ANOVA, *F*_1,22_ = 5.47, *p*<0.05; **Fig. 1A**). Performance significantly improved between the end of training on the first day and the retest on the second day in both groups, confirming that there was an offline improvement in performance on the trained sequence (*F*_1,22_ = 56.12, *p*<0.001). Supporting this, the planned ANOVA within each group indicated that both the 9- and 11-year-old groups performed better on retest than at the end of training (9-year-olds, *F*_1,13_ = 21.93, *p*<0.001; 11-year-olds, *F*_1,9_ = 40.98, *p*<0.001; **Fig. 1B**). There was no significant interaction (*F*_1,22_ = 0.86, *p* = 0.36), and the planned group comparisons showed that the degree of offline improvement did not significantly differ between the two groups (unpaired two-tailed t-test, *t*_22_ = −0.18, *p* = 0.86).

### The relationship between the degree of improvement and sleep duration

**Figure 2:**
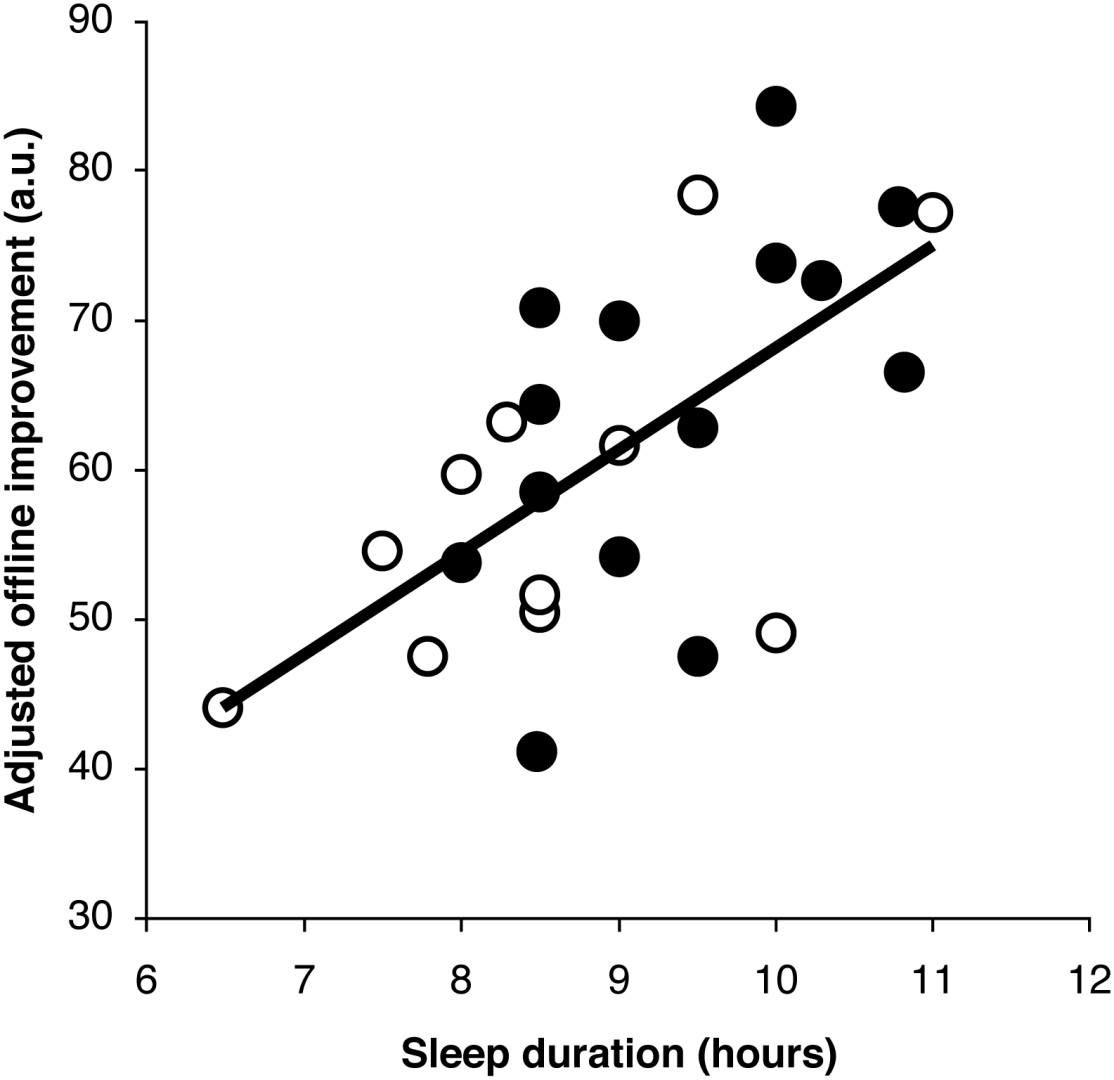
Sleep quantity and performance improvements. The degree of offline improvement, indicated by the performance improvement between testing days, significantly correlated with sleep duration the night after motor training (regression analysis; *β* = 0.65, *p*<0.05). The vertical axis represents the adjusted improvement in performance, ruling out the effects of age and the time between wake-up and testing on day 2, estimated from the results of the regression analysis. The horizontal axis indicates sleep duration (hours) during the night after motor training. The black and white dots represent the individual data from the 9- and 11-year-old children, respectively. The solid line is the linear regression fit.

Multiple regression analyses were conducted with the degree of improvement as the dependent variable and age, duration of sleep during the night after training (hours), and the time interval from wake-up to retest (hours) as independent variables (*R^2^* = .33). The total duration of sleep during the night after training had a significant positive effect on the degree of performance improvement (regression analysis, *β* = 0.60, *p*<0.01; **Fig. 2**). Neither age (*β* = 0.27, *p* = 0.18) nor time interval from wake-up (*β* = 0.24, *p* = 0.23) showed a significant relationship with improvement on the task.

To investigate the nature of the relationship between offline performance improvement and sleep duration, the fit of two non-linear models (quadratic and inverse models) were compared with that of the linear model. Model fitness did not differ between the linear and non-linear models: linear (*R^2^* = .22), quadratic (*R^2^* = .22, *p*<0.10), and inverse functions (*R^2^* = .22).

### Subjective ratings of sleepiness, concentration, and fatigue

The additional subjective ratings (sleepiness, concentration, and fatigue) did not significantly differ between two age groups or on the two testing days, and did not influence the degree of improvement on the task. We used an ANOVA to compare the subjective rating scores between groups (9- *vs.* 11-year-olds) and days (day 1 *vs.* day 2). There were no main effects for any of the rating scores (ANOVA, *p* values≥0.52). However, for the concentration rating, the interaction between group and days approached significance (*F*_1,22_ = 3.41, *p* = 0.08). There was no interaction for sleepiness or fatigue ratings (ANOVA, *p* values≥0.42). To factor in the effect of differences in concentration on the relationship between sleep duration and task improvement, the difference in concentration ratings between the days was added into the multiple regression analyses as an independent variable. The positive effect of sleep duration on the degree of improvement was still significant (regression analysis, *β* = 0.61, *p*<0.01).

## Discussion

Our behavioral data show that both the 9- and the 11-year-old groups of children showed novel skill acquisition following training on the sequential finger-tapping task on day 1, whereas the baseline skill levels significantly differed between the two age groups. Further, both age groups showed a significant improvement in performance during the night following motor training, confirming the results of previous investigations [11], [12]. Furthermore, the degree of this improvement was positively correlated with the duration of sleep the night after training. In this study, the length of the retention interval between training and retest was the same for all participants, while the number of hours slept during the retention interval varied for each individual. Taken together, the present observations demonstrate that in children, as in adults, the enhancement of motor sequence learning is associated with sleep rather than the simple passage of time.

According to the present data, the offline improvement in performance was linearly correlated with the number of hours of sleep during the night after training. Previous adult studies have demonstrated that enhanced performance on sequential motor tasks is associated with NREM 2 sleep and sleep spindles [8]–[10]. In normal human adults, NREM sleep and REM sleep alternate throughout the night with an approximately 90 minute cycle [22]. The number of cycles per night is normally distributed across individuals [23]. The amount of NREM 3 and 4 sleep decreases throughout the night, while REM and NREM 2 sleep increases [22]. Sleep spindles also increase over consecutive sleep cycles [24]. Furthermore, sleep parameters alter with age: in normal aging, the amount of NREM 3 and 4 sleep decreases while REM sleep increases [25]. In healthy children, the mean duration of the sleep cycle decreases with age, while the number of sleep cycles increases [26]. Despite the variability mentioned above, it is possible that a longer overall sleep duration is due to an increase in the number of sleep cycles, particularly within the same age group in children without any sleep problems. Thus, the amount of NREM 2 sleep may increase as overall sleep duration increases, due to an increase in the number of sleep cycles, resulting in enhanced consolidation.

Previous sleep-wake studies in children have shown that declarative memory in children benefits from sleep, but skill consolidation does not [13]–[15], which is inconsistent with the present results. This may be due to differences in the characteristics of the tasks, the age of the participants, or both. Fischer et al. [13] and Prehn-Kristensen et al. [15] both used an implicit motor learning task, whereas we utilized an explicit sequential finger-tapping task, which showed sleep-related consolidation in adults [8], [17], [27]. The benefit of sleep on implicit motor training in adults has been controversial [6], [7], [28], [29]. However, Fischer et al. [13] found the sleep-dependent deterioration in measures of implicit sequence knowledge in 7 to 13 year-old children in contrast to the gain of such knowledge in the adults during sleep. They concluded that the functional role of sleep in implicit memory consolidation depends on age. Our present finding is that sleep duration was correlated with the rate of offline performance improvement of explicit sequence learning in 9 and 11 year-old groups, suggesting the skill enhancement for the explicit motor learning is sleep-associated as in adults. Therefore, the age-dependency of sleep effect may be task dependent.

An alternative explanation for the present results is that participants who had less sleep during the night following training were more tired at the retest, resulting in poorer performance. However, all participants slept for at least 6 hours, and there was no correlation between sleep duration and the level of fatigue at retest (*r* = .33, **Table 1**). These data suggest that sleep duration was independent from the subjective ratings of fatigue. Also, we explicitly modeled the time intervals between waking and retest in the multiple-regression analysis to control for the possibility that participants who had been awake for a longer time period were more tired during retest. Thus, our present findings show that sleep during the night following training is indeed associated with skill enhancement in these age groups.

### Limitations of the study

The present study has some limitations that should be considered. First, we focused on explicit motor sequence learning, in which previous adult studies have demonstrated robust sleep-dependent consolidation [8], [17], [27]. In contrast, it is not clear whether consolidation on motor adaptation tasks, such as force-field adaptation, depends on sleep or the passage of time [3], [7], [28], [29]. Therefore, future investigations should determine whether the consolidation of motor adaptation in children depends on sleep or time.

Second, we did not include a control group that did not sleep, which is important to demonstrate the causal relationship between sleep and skill enhancement. We omitted this control because our hypothesis was based on adult studies that included an awake control group, and which established the impact of NREM 2 sleep on the offline improvement of explicit sequential motor skills [8], [9]. Future studies should demonstrate the causal role of sleep on skill enhancement in children older than 9 years by comparing sleep and wake groups, as has been shown in adults.

Third, the duration of sleep following motor sequence learning was observed and recorded by each participant’s parents, and was not directly measured. As these recorded sleep durations also included sleep latency, our measure of sleep duration is more or less equivalent to the self-report measures that are frequently used in adult sleep studies. Even though this measure is only an estimate and is subjective, a previous adult study showed a significant correlation between total sleep duration and the degree of offline performance improvement in perceptual skills using subjective sleep reports [30]. Therefore, the present sleep data is reliable to a certain extent, but future studies using polysomnography in children are warranted in order to clarify the relationship between specific sleep architectures, such as NREM 2 sleep, and the rate of skill enhancement during sleep following skill training.

In summary, the present study showed that sleep duration after motor sequence leaning is positively correlated with offline performance improvement in 9- and 11-year-old children. Therefore, we conclude that sleep is associated with skill enhancement for explicit motor sequence learning in children as well as in adults. Given that children are involved in a great deal of skill training in everyday life, understanding the mechanisms of motor skill learning in children might contribute to improved school performance, and also inform educational and welfare programs.

## Supporting Information

File S1 Contains the following files: **Table S1.** Demographic data and Subjective ratings (*n* = 25). **Table S2.** Individual skill performances in each trial (*n* = 25).
(PDF)
