# Supplementary material for: Sleep Is Associated with Offline Improvement of Motor Sequence Skill in Children
Source: PLoS One. 2014 Nov 5;9(11):e111635. doi: 10.1371/journal.pone.0111635 (PMC4221057; doi:10.1371/journal.pone.0111635)
Supplement: File S1 — Contains the following files: Table S1. Demographic data and Subjective ratings (n = 25). Table S2. Individual skill performances in each trial (n = 25). (PDF) [file pone.0111635.s001.pdf]

Table S1. Demographic data and Subjective ratings (*n* = 25)

ID	Time	Age	Gender	Handedness	Sleepiness day1 (1 - 10)	Concentration day1 (1 - 10)	Fatigue day1 (1 - 10)	Sleep onset day1	Sleep offset day1	Sleepiness day2 (1 - 10)	Concentration day2 (1 - 10)	Fatigue day2 (1 - 10)	Sleep onset day2	Sleep offset day2
1	15:00	11	female	0.33	1	10	10	22:40	6:40	1	10	10	22:00	6:20
2	9:00	8	female	1.00	10	10	10	21:30	6:00	10	10	10	21:00	6:00
3	9:00	9	male	0.82	8	9	6	22:00	7:30	9	9	8	21:30	6:00
4	10:50	10	female	0.94	9	10	8	23:00	5:30	10	9	9	22:30	6:00
5	16:00	10	female	1.00	9	10	10	21:30	6:00	8	10	7	22:30	6:20
6	9:00	11	female	-1.00	1	10	1	22:30	6:30	10	10	10	21:30	4:00
7	10:50	8	female	1.00	9	10	8	21:00	6:00	10	9	10	20:30	7:15
8	13:00	10	male	1.00	9	9	10	22:00	7:30	10	9	10	21:20	7:30
9	14:50	8	male	0.93	6	10	7	22:00	6:00	8	10	5	21:00	7:00
10	16:00	11	male	1.00	3	8	7	22:30	6:30	1	9	5	22:00	6:30
11	10:50	11	male	1.00	9	8	8	21:15	7:00	8	7	8	23:00	7:00
12	12:00	9	male	1.00	9	10	10	23:00	7:00	9	10	10	21:30	7:00
13	16:00	8	male	1.00	9	5	8	21:30	6:30	3	4	5	21:30	6:00
14	9:00	9	female	0.94	5	5	10	21:30	6:00	5	8	8	20:45	7:00
15	10:50	9	male	1.00	10	9	10	22:00	7:30	10	8	9	21:30	7:00
16	13:00	11	male	0.88	10	10	9	22:00	6:00	8	10	10	21:30	7:00
17	14:50	9	male	1.00	5	6	4	22:00	6:00	9	10	9	21:00	6:00
18	17:00	9	male	0.89	5	10	10	21:15	8:00	5	10	10	21:15	8:00
19	9:00	11	female	0.90	9	10	10	22:00	8:00	10	10	9	21:00	8:00
20	10:50	11	male	0.89	4	9	10	21:30	6:00	7	7	10	21:30	6:00
21	16:00	8	male	0.00	7	6	8	21:20	7:30	5	7	6	21:00	7:00
22	10:50	8	female	0.93	5	9	3	23:00	6:00	9	10	6	21:30	6:00
23	13:00	9	female	1.00	5	6	4	21:00	6:00	3	6	5	21:30	6:00
24	14:50	9	female	0.89	5	8	6	22:00	6:00	5	8	4	22:00	6:00
25	16:00	10	male	1.00	6	10	8	22:20	7:20	4	7	6	22:30	7:30

Table S2. Individual skill performances in each trial (*n* = 25)

ID	Training 1	Training 2	Training 3	Training 4	Training 5	Training 6	Training 7	Training 8	Training 9	Training 10	Training 11	Training 12	Test 1	Test 2	Test 3	Test 4	Test 5
1	17.20	19.00	20.60	20.80	19.80	19.40	21.20	21.60	21.20	19.40	20.00	18.40	24.80	23.80	24.00	22.80	23.20
2	5.80	5.20	6.20	6.00	6.40	6.00	5.80	6.60	8.20	8.60	9.40	9.60	10.80	10.60	11.20	11.40	11.80
3	10.00	10.20	12.20	12.80	12.00	14.20	14.40	14.20	14.60	14.80	14.40	15.20	18.80	17.40	17.80	16.80	16.20
4	12.60	16.60	17.60	19.80	20.40	19.40	19.60	20.40	23.80	24.00	23.20	24.00	25.40	26.00	26.80	27.60	27.40
5	12.80	14.40	16.20	18.20	21.00	19.00	18.60	20.20	20.40	18.00	20.40	20.40	21.20	19.40	22.80	23.80	23.40
6	12.00	14.60	16.80	15.40	14.60	15.60	15.80	17.60	17.60	18.40	19.40	18.60	20.00	18.40	19.40	19.60	20.00
7	9.00	8.40	8.60	7.80	8.80	10.20	8.60	9.40	8.40	9.80	10.40	10.60	11.20	12.40	11.80	12.80	12.60
8	9.80	11.20	13.60	13.40	13.00	14.40	15.00	15.60	14.80	15.60	16.80	16.80	16.60	16.60	18.60	17.60	16.40
9	10.00	9.60	9.40	10.20	9.60	11.60	12.60	12.00	14.20	11.20	14.80	14.40	19.60	18.20	17.60	17.20	18.00
10	17.20	19.80	22.00	21.20	21.00	21.60	18.60	20.20	19.60	22.80	23.60	21.60	27.40	24.60	25.00	25.20	25.20
11	9.20	10.20	12.60	12.80	11.60	13.40	12.60	11.80	12.20	15.00	12.60	14.40	17.80	14.80	16.40	16.60	17.40
12	11.80	10.60	12.20	13.20	13.20	13.20	14.00	15.60	14.00	15.00	16.00	15.40	15.20	15.80	15.60	13.80	17.20
13	11.40	13.00	12.80	11.20	10.80	12.60	14.80	13.80	14.60	13.20	15.00	13.60	17.60	13.60	16.40	17.60	17.80
14	10.00	10.20	10.60	12.20	14.80	15.00	16.20	17.60	18.00	20.60	19.60	19.40	25.40	24.80	23.00	21.20	24.20
15	9.20	9.40	10.60	11.60	13.60	12.60	12.40	11.40	12.80	12.40	13.40	11.00	14.00	13.80	14.20	14.00	14.00
16	10.00	13.20	11.40	11.40	12.40	12.00	10.80	12.40	12.40	13.20	12.20	12.20	18.20	17.40	16.20	14.40	15.40
17	9.80	11.80	12.40	11.60	11.20	11.80	12.20	13.20	13.20	12.80	13.20	13.60	15.20	15.00	13.60	13.40	11.80
18	8.80	8.40	9.20	9.40	9.60	8.60	10.60	10.60	10.40	10.60	10.60	10.20	14.40	14.40	13.40	12.20	12.60
19	9.20	10.60	9.60	11.80	12.80	12.20	12.60	13.00	12.00	13.20	12.20	11.40	16.20	15.60	16.60	14.80	16.20
20	11.80	13.20	14.40	14.20	16.00	15.20	15.80	15.60	15.60	17.60	17.00	19.20	20.20	19.80	18.80	19.00	19.80
21	9.20	9.00	8.80	9.40	9.00	9.80	9.00	9.60	9.20	11.00	10.80	10.80	13.40	13.20	15.20	14.00	12.60
22	9.60	9.20	12.20	14.60	15.00	18.60	14.60	16.60	18.20	18.40	19.20	19.00	18.00	17.80	15.80	16.20	20.00
23	9.20	9.00	9.40	10.00	10.20	10.20	9.60	9.80	12.40	10.80	13.00	13.80	15.40	15.00	14.40	14.80	14.60
24	11.80	14.60	14.40	14.60	16.20	15.20	15.60	16.60	14.20	15.80	16.80	18.00	20.00	17.00	18.60	15.20	20.00
25	8.20	13.20	11.80	13.60	13.20	14.40	13.20	14.40	12.20	12.40	10.60	11.20	14.00	13.80	13.40	16.00	15.80
